# A hemodialysis patient with recurrent Wernicke encephalopathy showed reversible lentiform fork sign: A case report

**DOI:** 10.1097/MD.0000000000047911

**Published:** 2026-03-20

**Authors:** Wencong Liang, Yueyao Chen, Yaochi Zeng, Shudong Yang

**Affiliations:** a Department of Nephrology, Shenzhen Traditional Chinese Medicine Hospital, The Fourth Clinical Medical College of Guangzhou University of Chinese Medicine, Shenzhen, Guangdong, China; b Department of Radiology, Shenzhen Traditional Chinese Medicine Hospital, The Fourth Clinical Medical College of Guangzhou University of Chinese Medicine, Shenzhen, Guangdong, China; c Department of Nutrition, Shenzhen Traditional Chinese Medicine Hospital, The Fourth Clinical Medical College of Guangzhou University of Chinese Medicine, Shenzhen, Guangdong, China.

**Keywords:** case report, hemodialysis, lentiform fork sign, thiamine deficiency, Wernicke encephalopathy

## Abstract

**Rationale:**

This case report describes an unusual presentation of recurrent Wernicke encephalopathy (WE) in a patient undergoing hemodialysis, with distinctive neuroimaging findings that provide new insights into the disease.

**Patient concerns:**

The patient presented with progressive gait ataxia, dysarthria, and mild memory impairment, with recurrent neurological symptoms following thiamin discontinuation.

**Diagnoses:**

WE was diagnosed based on characteristic MRI findings including the rare lentiform fork sign and other atypical brain lesions, supported by symptom recurrence after thiamin cessation and radiological reversibility with treatment.

**Interventions:**

The patient received immediate thiamin supplementation therapy, close neurological monitoring, and regular hemodialysis.

**Outcomes:**

Neurological symptoms significantly improved with treatment, and MRI showed reversal of the lentiform fork sign and other atypical lesions, demonstrating the importance of sustained thiamin management during follow-up.

**Lessons:**

This case highlights the lentiform fork sign as a valuable neuroimaging marker for WE, suggests that blood–brain barrier dysfunction may be pathogenic, and emphasizes the need for continuous thiamin management in high-risk patients to prevent recurrence.

## 1. Introduction

Wernicke encephalopathy (WE) is an acute neurological complication caused by thiamin deficiency. The clinical manifestations of WE include ophthalmoplegia, gait ataxia, and neurological disorders, such as memory impairment.^[1]^ The mortality rate from WE is as high as 20%,^[2]^ but clinically, it is prone to underdiagnosis.^[3,4]^ In the diagnosis of WE, MRI has a sensitivity of 53% but a specificity of 93%.^[5]^

The characteristic manifestations of WE are bilateral, symmetrical hyperintense signals in the mammillary bodies, thalamus, 3rd ventricle and midbrain periaqueductal area on brain MRI.^[6,7]^ Basal ganglia lesions are very rare in WE. Basal ganglia lesions, especially the lentiform fork sign, are specific MRI findings for the early diagnosis of uremic encephalopathy (UE).^[8–10]^ We herein describe a patient on hemodialysis who experienced a recurrence of WE due to the discontinuation of thiamin. When the patient exhibited gait ataxia, we noted the possibility of WE, and thiamin supplementation improved the patient’s condition. However, after discontinuation of thiamin, the patient’s clinical symptoms unfortunately recurred. The radiologically reversible findings, including the lentiform fork sign, were consistent with the treatment response.

## 2. Case presentation

A 53-year-old male without a history of alcoholism with a high school diploma usually makes a living as a security guard. In April 2022, the patient was diagnosed with uremia secondary to diabetic nephropathy and received maintenance hemodialysis. He was hospitalized because of gait ataxia, dysarthria, and mild memory impairment on December 9, 2022.

The patient had a 10-year history of diabetes; however, he was not currently taking hypoglycemic medication. His glycated hemoglobin A1c level was 5.9% (normal reference range: 4.0–6.0%), indicating well-controlled blood glucose levels. Additionally, the patient had a 10-year history of hypertension and was currently taking oral antihypertensive medication, specifically metoprolol succinate sustained-release tablets (47.5 mg, once daily) and nifedipine controlled-release tablets (30 mg, once daily). The patient’s blood pressure remaines well-controlled. He did not take any other medications that could affect cognitive function or cause organic diseases of the central nervous system. The patient denied any family disease history or genetic disease history.

### 2.1. First hospitalization

#### 2.1.1. General examination

Consciousness was clear, but speech was slurred, and an ataxic gait was present. Orientation and attention were diminished, and digital ability and memory were impaired. Height was 170.0 cm, weight was 59.1 kg, and body mass index was 20.45. Normal food intake had decreased by more than 50%, and weight loss over the past 3 months had exceeded 10 kg (>15%). Blood pressure was 166/90 mm Hg. Pulse: 61 beats per minute. Temperature: 36.6°C.

Muscle tone had increased. The level of muscle strength was 5. There was no bundle tremor. There was no involuntary limb movement. Pain and touch sensations, as well as complex sensations, were normal bilaterally. Tendon reflexes are normal. There were no bilateral pathological signs (−).

#### 2.1.2. Diagnosis

**Figure 1.**
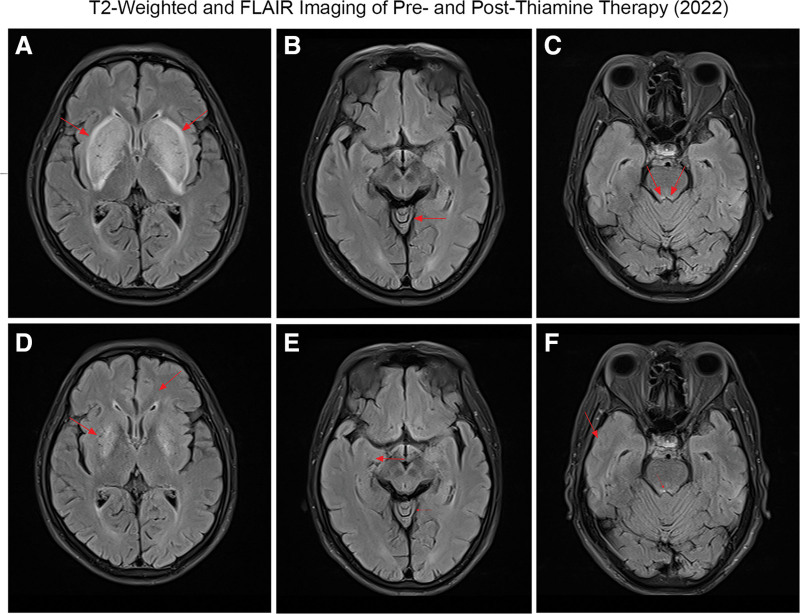
On the 1st hospitalization, red arrows indicated hyperintense signals in bilateral basal ganglia lesions, representing the “lentiform fork sign” (A), the cerebellar vermis (B), and the dorsal pons (C) on T2-weighted and fluid-attenuated inversion recovery (FLAIR) images. After thiamin therapy, hyperintense signals were decreased in bilateral basal ganglia lesions (D), the cerebellar vermis (E), and the dorsal pons (F) on T2-weighted and FLAIR images.

He developed cerebellar dysfunction manifested as ataxia, along with mild memory loss. Significant weight loss and dietary deficiencies were noted. On December 9, 2022, the patient underwent the 1st cranial magnetic resonance examination (Fig. 1). The thiamin concentration in the serum was 0.97 ng/mL (the reference value range was 1.7–10 ng/mL). WE was considered in accordance with the EFNS guidelines for WE.^[1]^

#### 2.1.3. Therapeutic interventions

We continued with the current hemodialysis protocol. The patient received intramuscular injections of 300 mg of thiamin twice daily, totaling 600 mg per day.^[11]^ After thiamin supplementation, the thiamin concentration was 2539.27 ng/mL, and the patient continued taking 30 mg of thiamin tablets daily.

#### 2.1.4. Follow-up

The patient’s memory improved, ataxia resolved, and he was able to walk alone, but dysarthria persisted. MRI showed reversal of the lentiform fork sign and other atypical lesions (Fig. 1). The patient was subsequently discharged. However, beginning on January 24, 2023, the patient did not follow medical advice and stopped taking thiamin tablets.

### 2.2. Second hospitalization

In March 2023, the patient experienced a recurrence of gait ataxia, dysarthria, and memory loss, as shown in Supplemental Digital Content 1 (Video 1); subsequently, the patient was admitted to the hospital.

**Video 1.** 

#### 2.2.1. General examination

His orientation, attention, and computational abilities, as well as his recall ability, were all reduced to varying degrees. The Mini-Mental State Examination (MMSE) score was 23 (with the MMSE cutoff set at 21 based on the patient’s education level), indicating mild cognitive impairment.

The contents of trace elements, including lead, magnesium, zinc, copper, iron, and calcium, were within normal ranges. Body composition analysis revealed muscle deficiency in the patient. The EEG was normal, whereas electromyography suggested peripheral neuropathy. Genetic testing did not reveal any genetic variants associated with the clinical manifestations. The relevant laboratory results before and after hemodialysis suggested an adequate hemodialysis effect.

#### 2.2.2. Diagnosis

**Figure 2.**
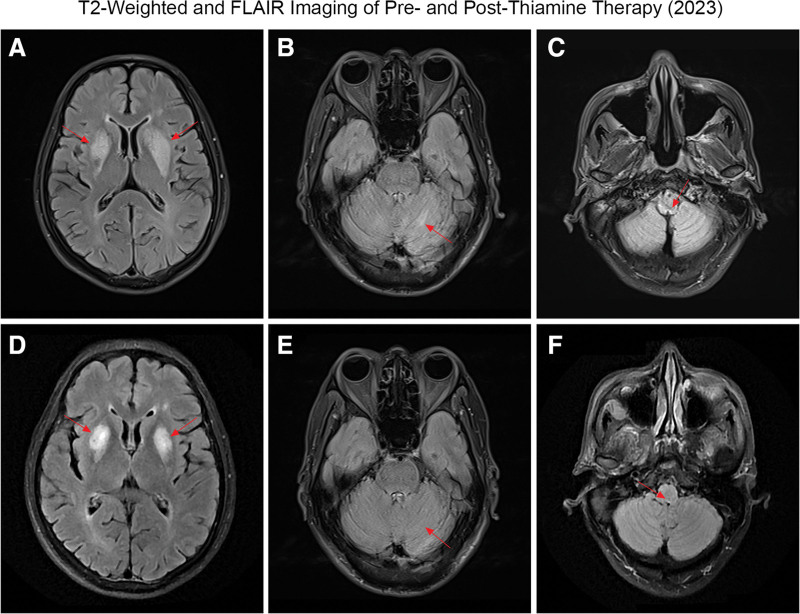
After thiamin cessation, MRI revealed that the bilateral basal ganglia lesions had progressed compared with the 1st hospitalization. Red arrows indicated hyperintense signals in bilateral basal ganglia lesions (A), the cerebellar dentate nucleus (B), and the dorsal medulla (C). After thiamin supplementation, hyperintense signals were decreased in bilateral basal ganglia lesions (D), the cerebellar dentate nucleus (E) and the dorsal medulla (F) on T2-weighted and FLAIR images. FLAIR = fluid-attenuated inversion recovery.

The patient again experienced ataxia, dysarthria, and mild memory impairment. MRI revealed that the bilateral basal ganglia lesions had progressed compared with those at the 1st hospitalization (Fig. 2). The thiamin concentration in the serum was 2.24 ng/mL. Blood gas analysis was performed prior to hemodialysis, revealing a pH value of 7.36 (reference range: 7.35–7.45) and a lactate concentration of 1.9 mmol/L (reference range: 0.5–2.0 mmol/L). Simultaneously, the patient’s blood glucose level was 4.67 mmol/L, ruling out diabetic ketoacidosis and lactic acidosis. The patient underwent hemodialysis 3 times a week, with a minimal risk of developing UE. He was not taking metformin; owing to detection limitations, toxicological screening was not conducted. Thiamine treatment promptly alleviated the symptoms, serving as a crucial diagnostic criterion for WE. We believe that the cessation of thiamin for nearly 2 months led to the recurrence of WE.

#### 2.2.3. Therapeutic interventions

We continued with the current hemodialysis protocol. The patient received intramuscular injections of 300 mg of thiamin twice daily, totaling 600 mg per day. After thiamin treatment, the serum thiamin concentration was 515.74 ng/mL, and the patient continued taking 30 mg of thiamin tablets daily.

#### 2.2.4. Follow-up

**Figure 3.**
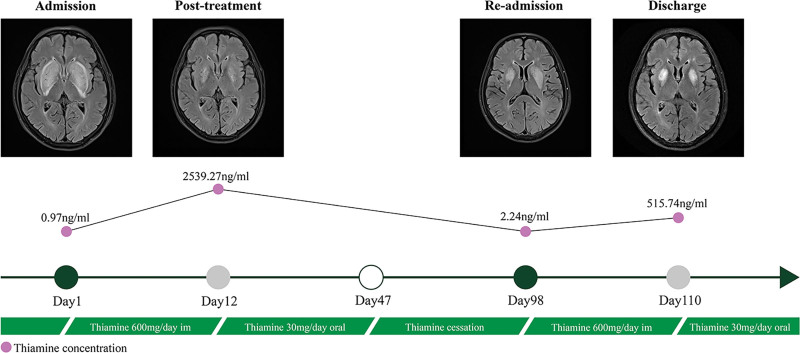
The timeline of the clinical process is depicted.

His ataxia disappeared, as shown in Supplemental Digital Content 2 (Video 2), the retest MMSE score was 28 (out of a total score of 30), and he was able to live on his own. MRI revealed that the lesions, such as those in the bilateral basal ganglia, had again improved (Fig. 2). The timeline of the clinical process is depicted in Figure 3.

**Video 2.** 

The patient had been taking oral thiamin supplements but had not adhered to regular follow-up tests to monitor thiamin levels. At the 1-year telephone follow-up post-discharge, the patient had no gait ataxia but presented with mild dysarthria.

## 3. Discussion

The case report details the patient’s 2 clinical courses, including the 1st onset of illness and relapse after thiamin discontinuation, and the laboratory and imaging data are complete. Overlapping imaging characteristics on MRI are very rare, and the lentiform fork sign, as an atypical manifestation, is an important supplement to the neuroimaging findings of WE, which can lead to an earlier diagnosis of WE and may also serve as a prognostic indicator. These findings could help clinicians assess the effectiveness of treatment.

For patients undergoing hemodialysis, regular monitoring of thiamin levels is crucial. Patients with chronic kidney disease are at risk of malnutrition. Uremic toxins damage the gastrointestinal mucosa, reducing thiamin uptake and absorption. The dialysis process can easily cause the loss of water-soluble substances, especially thiamin,^[12,13]^ which can damage brain energy metabolism. Studies have confirmed that 10% of hemodialysis patients are thiamin-deficient, and that prompt thiamin supplementation effectively alleviates neurological dysfunction.^[14]^ If we consider the lentiform fork sign solely as a manifestation of uremia and intensify hemodialysis to clear toxins, treatment for patients can aggravate their thiamin deficiency, eventually leading to the development of more severe neurological diseases, such as Korsakoff syndrome. The clinical presentation of Wernicke–Korsakoff syndrome is not related to alcoholism.^[15]^ However, in nonalcoholic patients, some atypical MRI findings could include basal ganglia lesions.^[16–19]^ We also noted that changes in reversible high signals on brain MRI are related to the dosage of thiamin supplementation.^[20]^

We have summarized the cases presenting with the lentiform fork sign on MRI over the past 3-years, as depicted in Table 1.^[21–26]^ The lentiform fork sign is observed in patients with lactic acidosis. Patients are advised to undergo a serum thiamin concentration test, as thiamin deficiency can lead to severe lactic acidosis.^[27–29]^

**Table 1:** Summary of the cases presenting with the lentiform fork sign on MRI.

Reference source	Patient information	Clinical manifestations	Diagnosis	Treatment
Correia, C. et al^[21]^	A 70-yr-old woman with type 2 diabetes and chronic kidney disease on hemodialysis	Acute onset chorea	Uremic encephalopathy	Olanzapine and hemodialysis 3 times per week.
Ben Lakhal, A. et al^[22]^	An 11-yr-old boy	Pseudobulbar syndrome and parkinsonism	Post-mumps encephalitis	Steroid treatment and therapeutic plasma exchange.
Santos, MA. et al^[23]^	A 58-yr-old woman with complications of diabetic nephropathy, on dialysis	Restlessness, dizziness, slurred speech, and ataxic gait	Metformin-induced encephalopathy	Hemodialysis and discontinuation of metformin.
Albadr, F. et al^[24]^	A 56-yr-old male with a history of chronic kidney disease and diabetes mellitus	Slurred speech, left-sided weakness, and dysarthria	Uremic encephalopathy	Metformin was discontinued and hemodialysis.
Yang, Y. et al^[25]^	A 38-yr-old man	Coma and visual disturbances	Methanol poisoning	Mechanically ventilated and the metabolic acidosis was corrected.
Alhusseini, A. et al^[26]^	A 56-yr-old male with end-stage renal disease	A progressive change in mental status and involuntary arm movements	Extrapyramidal syndromes of chronic kidney disease and dialysis	Intensified hemodialysis and glycemic control.

Therefore, how does thiamin deficiency cause the lentiform fork sign? In the human body, the active form of thiamin is thiamin pyrophosphate, which is an indispensable coenzyme factor in the tricarboxylic acid cycle. Thiamin pyrophosphate plays a crucial role in the catalytic reactions of various key enzymes, including pyruvate dehydrogenase, α-ketoglutarate dehydrogenase, and transketolase. Thiamine plays a crucial role in the metabolism of glucose and in sustaining the proper function of nerve cells. Thiamine deficiency results in alterations in glucose homeostasis and neurodegenerative lesions. Thiamine deficiency can lead to the conversion of most glucose into lactic acid and pyruvate.^[30]^ It causes lactic acidosis in neuronal cells and energy failure.^[31]^ Metabolic acidosis often occurs in patients with WE.^[32]^ Thiamine deficiency results in mitochondrial dysfunction,^[33]^ which in turn causes abnormalities in brain energy metabolism. These abnormalities include oxidative stress, inflammation, and excitotoxicity, all of which contribute to neuronal cell death.^[34,35]^ Astrocytes are important components of the blood–brain barrier, and some studies suggest that astrocytes are the main target of WE.^[36,37]^ Thiamine deficiency results in the production of reactive oxygen species and various cytokines, leading to astrocyte dysfunction. Meanwhile, lactic acidosis impairs astrocyte glutamate uptake; glutamate accumulation leads to excitotoxicity in neurons, ultimately resulting in neuronal death.^[38,39]^ The blood–brain barrier is damaged and progresses to vasogenic edema,^[35]^ so cranial MRI may reveal hyperintense signals in the basal ganglia, which is the lentiform fork sign.

The patient we encountered was a nonalcoholic dialysis patient who found thiamin treatment very safe and effective. As demonstrated in the video, after thiamin supplementation, his ataxic gait disappeared rapidly. The reversibility of radiological abnormalities following thiamin supplementation therapy underscores the importance of timely intervention. Moreover, the recurrence of the patient’s condition after the discontinuation of thiamin supplementation highlights the crucial role of ongoing treatment in managing WE. Therefore, for WE, early diagnosis and continuous treatment should be pursued to prevent further neurological deterioration.

Nutritional support and alcohol withdrawal are essential for the rehabilitation of patients with WE. Nicotine exposure significantly inhibits thiamin uptake in pancreatic acinar cells.^[40]^ Thiamin is naturally found in whole grains, legumes, fish, and lean meat, while present in relatively low concentrations in dairy products, fruits, and vegetables. Tannic acid, present in betel nuts and tea, inhibits thiamin absorption.^[41]^ The heat-resistant thiaminase found in silkworm larvae depletes thiamin in the human body, thereby causing acute ataxia.^[42]^ Low-carbohydrate diets decrease thiamin intake and thiamin diphosphate levels.^[43]^ Patients are advised to avoid ultra-processed foods and beverages, as well as a ketogenic diet, because case reports suggest that a ketogenic diet can exacerbate WE.^[44–46]^ High-temperature cooking should be avoided since thiamin is water-soluble and heat-sensitive. We assessed patients’ specific dietary habits during regular phone calls and outpatient visits. Regrettably, for a patient living alone, adherence to the recommended dietary plan remains low. The patient had no history of alcohol consumption and never consumed nuts to prevent hyperkalemia. Owing to infrequent home cooking, he relied on fast food, such as steamed fish with white rice and stir-fried lean meat set meals. His intake of fruit and vegetables was relatively low. The Kidney Disease Outcomes Quality Initiative guideline^[47]^ recommended that hemodialysis patients with inadequate dietary intake be provided with multivitamins including thiamin. Nevertheless, there are no unified or regulated standards for thiamin supplementation dosage or screening procedures in hemodialysis patients. Based on this case report, we consider it highly necessary to investigate the dietary intake of hemodialysis patients and to measure thiamin concentration, particularly in those with neurological symptoms or malnutrition.

Conventional MRI is highly valuable for diagnosing WE, but it has limitations: it cannot directly assess cerebral blood flow or metabolic status. We should explore the application of advanced MRI modalities, such as diffusion tensor imaging and perfusion studies, in patients with atypical WE. For example, diffusion tensor imaging can sensitively detect microstructural damage in the white matter of atypical WE patients. Lyu et al^[48]^ employed multimodal MRI to evaluate the pathophysiology of WE, confirming the pathophysiological sequence of “thiamin deficiency → cellular metabolic disorder → edema/inflammation → decreased blood perfusion.” Li et al^[49]^ discovered that arterial spin labeling detected increased cerebral blood flow in the lesion area; it can detect abnormal microcirculation in the brain prior to the identification of cytotoxic edema via diffusion-weighted imaging. A study indicated that an MRI review of WE patients post-thiamin treatment revealed an improvement in diffusion restriction, and cerebral blood flow in affected structures normalized.^[50]^ These findings suggest that arterial spin labeling could also serve as an imaging biomarker to assess the efficacy of thiamin treatment.

## 4. Conclusion

Patients presenting with neurological symptoms require immediate differentiation between WE and UE.^[51,52]^ Their distinct characteristics are detailed in Table 2. Notably, hyperintensities in the bilateral basal ganglia on T2/fluid-attenuated inversion recovery are one of the features of atypical WE.^[53,54]^ The lentiform fork sign is an important supplement to neuroimaging in WE. Proper and timely administration of thiamin aids in the restoration of radiological abnormalities. For high-risk patients, we emphasize the importance of continuous management to prevent recurrence.

**Table 2:** Summary of differentiating dimensions between WE and UE.

Differentiating dimensions	WE	UE
Core etiology	Thiamine deficiency	Toxin accumulation due to renal failure.
Clinical features	Confusion, ophthalmoplegia, ataxia	Asterixis, progressive loss of consciousness.
Key laboratory tests	Decreased serum thiamin level	Markedly elevated serum creatinine and urea nitrogen.
Typical MRI (T2/FLAIR)	Hyperintensities within the thalamus, mammillary bodies, and periaqueductal gray matter	Hyperintensities within the bilateral basal ganglia.
Atypical MRI (T2/FLAIR)	Hyperintensities within the bilateral basal ganglia, dentate nucleus, and cerebellar hemispheres	Hyperintensities within the brainstem, cerebellum, and hippocampus.
Treatment principles	Thiamine supplementation	Renal replacement therapy (dialysis).

FLAIR = fluid-attenuated inversion recovery; UE = uremic encephalopathy; WE = Wernicke encephalopathy.

## Acknowledgments

We would like to express our gratitude to the patient for granting permission to use their clinical data in this paper and for the publication of this research.

## Author contributions

**Data curation:** Wencong Liang, Yueyao Chen.

**Funding acquisition:** Shudong Yang.

**Investigation:** Yaochi Zeng.

**Supervision:** Shudong Yang.

**Writing – original draft:** Wencong Liang.

**Writing – review & editing:** Yueyao Chen, Yaochi Zeng, Shudong Yang.

## Supplementary Material


