# Supplementary material for: A hemodialysis patient with recurrent Wernicke encephalopathy showed reversible lentiform fork sign: A case report
Source: Medicine (Baltimore). 2026 Mar 20;105(12):e47911. doi: 10.1097/MD.0000000000047911 (PMC13008232; doi:10.1097/MD.0000000000047911)
Supplement: Supplementary file 3 [file medi-105-e47911-s003.pdf]

深圳市中医院医学伦理委员会
个案报道伦理审查申请及审批表

文章题目： 一名反复发作韦尼克脑病的血液透析患者出现可逆性豆状核叉征：病例报告

报道对象：（请描述） 血液透析患者 例数 1 例

申请科室： 肾病科 **申请人：** 梁文聪

递交审查资料：
☑个案报道文章 ☐知情同意书 ☐免除知情同意申请
☐其他资料________________

内容摘要：

本病例报告描述了一位血液透析患者反复发作的韦尼克脑病（Wernicke encephalopathy）的不寻常表现，其独特的神经影像学发现为疾病提供了新的见解。患者情况：患者表现为进行性步态共济失调、构音障碍和轻度记忆减退，在停用硫胺素后出现反复的神经系统症状。诊断：基于特征性的 MRI 发现，包括罕见的豆状核叉征和其他非典型脑病变，以及硫胺素停药后症状复发和治疗后影像学可逆性，诊断为 WE。干预措施：患者立即接受了硫胺素补充治疗、密切的神经监测以及规律的血液透析。结果：经过治疗，患者神经系统症状显著改善，MRI 显示豆状核叉征和其他非典型病变逆转，证明了随访期间持续硫胺素管理的重要性。

目的意义：

本病例强调了豆状核叉征作为韦尼克脑病有价值的神经影像学标记，提示血脑屏障功能障碍可能是致病因素，并强调了高风险患者需要持续的硫胺素管理以防止复发。

报道对象隐私保护措施：

对患者临床资料及个人信息负有保密义务和责任，所采集的数据仅在科学杂志发表进行学术讨论，绝不暴露或泄露患者个人信息，尊重患者隐私权，文章中不披露患者姓名、住院号、登记号、检查号、医保卡号、手机号等个人信息，依照国家相关法律法规要求进行去标识化处理。

申请人承诺：

以上所填内容均属实，严格遵守“尊重、有利/不伤害、公正”的伦理学原则和国际国内科研伦理规范。

签名： 梁文聪 梁文聪 日期：2025.5.10

伦理审查意见：

主任/副主任委员签字：[illegible]

深圳市中医院医学伦理委员会（盖章）

日期 2025 年 5 月 1 日

Patient Consent Form for Case Report

This consent form is provided by the authors to the patient himself/guardian/relative of the case report,and the signature indicates knowledge and consent that this paper will be published in a medical journal with patient related information.

Title of article: A hemodialysis patient with recurrent Wernicke encephalopathy showed reversible lentiform fork sign:A case report

Full Author: Wencong Liang,Yueyao Chen,Yaochi Zeng.

Corresponding author: Shudong Yang

To be completed by the patient:

I(Shangjun Zhou)give my consent for all or any part of this material to appear in the medical journal and associated publications.

Please check the box in front of the one of the two below that applies:

☑The author of the above article has explained to me in detail what the article is about.

☑ 1 have read the relevant information submitted to the magazine.

I fully understand and appreciate the following:

1.My(or the patient's)privacy is adequately protected in the article,and information related to my (or the patient's)personal identity and photographs,images,and text that identify the individual will not be disclosed.I know and understand that despite this,my(or the patient's)relative and the treating healthcare team may still associate me(or the patient)from the content of the article.

2.This information will be posted on the journal's website and relevant specialized databases.

3.Publication of this information is open to the entire world,and the journal's readership is primarily medical personnel,but non-physician members of the public,including journalists,may also have access to the information.

4.Journals will not use this information for commercial purposes such as advertising, packaging,or abridged use.

5.Iam able to withdraw my consent at any time prior to publication.This consent cannot be withdrawn once the information has been delivered for publication.

Signature : Shangjun Zhou Date: 2025-01-20

Author name: Wencong Liang Date: 2025-01-20
